# Epilepsy Cured by Use of Bromide of Potassium and Sulphate of Atropia

**Published:** 1872-12

**Authors:** 


Epilepsy Cured by the Use of Bromide of Potassium and Sulphate of Atropia.—Dr. L. P. Yandell, Jr., (*The American Practitioner*, September, 1872,) reports three cases of the above disease. In the first, a girl thirteen years of age, the bromide alone had been used in full doses for many months without relief. Dr. Y. gave her twenty-six grains of the bromide three times a day, and one hundredth of a grain of the sulphate of atropia night and morning. The attacks soon became less frequent, and in seven months had apparently disappeared. She was directed, however, to continue the medicine twelve months more.

